# Herbal medicine used in the treatment of cardiovascular diseases in the Rif, North of Morocco

**DOI:** 10.3389/fphar.2022.921918

**Published:** 2022-08-11

**Authors:** Noureddine Chaachouay, Abdelhamid Azeroual, Bouchaib Bencharki, Lahcen Zidane

**Affiliations:** ^1^ Interdisciplinary Research Laboratory in the Sciences, Education, and Training Indian Railways Institute Of Signal Engineering and Telecommunications (IRLSET), Hassan First University, Settat, Morocco; ^2^ Agri-Food and Health Laboratory (AFHL), Faculty of Sciences and Techniques of Settat, Hassan First University, Settat, Morocco; ^3^ Plant, Animal Productions and Agro-industry Laboratory, Department of Biology, Faculty of Sciences, Ibn Tofail University, Kenitra, Morocco

**Keywords:** cardiovascular diseases, herbal medicine, ethnobotany, ethnomedicine, medicinal and aromatic plant

## Abstract

**Background:** Since the dawn of time, Moroccans have used medicinal plants as a popular remedy to treat a wide range of human and cattle health issues. Nonetheless, very little research has been conducted in the past to record and disseminate indigenous ethnopharmacological knowledge adequately. This study was conducted in the Rif and attempted to identify medicinal plants used by indigenous people to treat cardiovascular problems and the ethnomedicinal knowledge linked with them.

**Methods:** The ethnobotanical study was carried out in the Moroccan Rif area over 2 years, from 2016 to 2018. We questioned 520 traditional herbalists and consumers of these herbs in total. The gathered data were examined and contrasted using quantitative ethnobotanical indicators such as family importance value (FIV), the relative frequency of citation (RFC), plant part value (PPV), fidelity level (FL), and informant consensus factor (ICF).

**Results:** The findings analysis revealed the presence of 33 plant species classified into 20 families, with the Poaceae dominating (7 species). Regarding disorders treated, the category of cardiac arrhythmias has the greatest ICF (0.98). The study discovered that the leaves were the most often utilized portion of the plants (PPV = 0.353) and that the most frequently used preparation was a decoction (31%).

**Conclusions:** The current study’s findings revealed the presence of indigenous ethnomedicinal knowledge of medicinal plants in the Moroccan Rif to treat cardiovascular illnesses. Further phytochemical, pharmacological, and toxicological investigations should be conducted to identify novel drugs from these documented medicinal plants.

## GRAPHICAL ABSTRACT

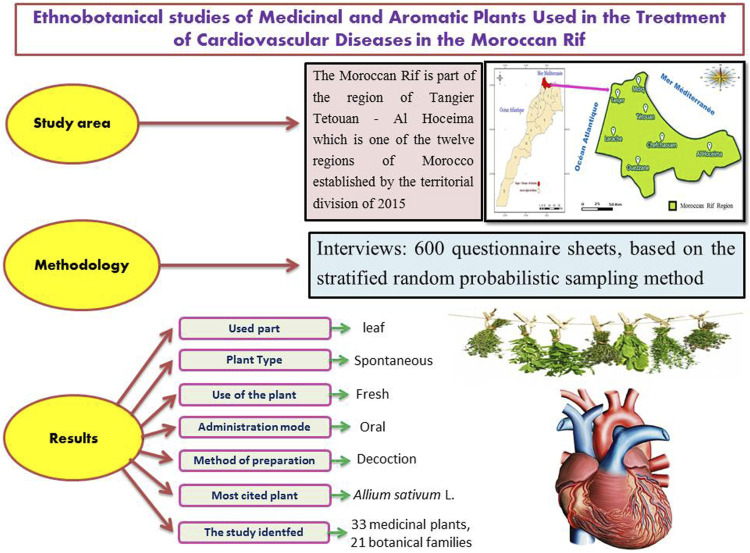

## 1 Introduction

Cardiovascular disorder (CVD) is the leading cause of mortality globally (Muna, 1993; Lopez et al., 2006; Strong et al., 2006). CVD encompasses many problems, including cardiac muscle diseases and the vascular system that supplies the heart, brain, and other essential organs. Humans have long utilized medicinal and aromatic plants (MAPs) to cure and combat illness. Traces of its usage may be found in all ancient civilizations and continents. Thus, despite advances in pharmacology, the medicinal use of plants is still prevalent in certain countries, particularly in impoverished nations (Gurib-Fakim, 2009).

In recent decades, plants have become essential sources of both preventative and therapeutic traditional medicinal preparations for humans and animals. Plants are also significant in global business nowadays (Zhang, 1998). In the lack of an excellent medical system, the demand for medicinal plants increases daily in underdeveloped and industrialized countries (World Health Organization, 2001).

Due to its relief and biogeographical location, Moroccan Rif has a vibrant ecological, and floristic diversity, becoming a natural plant genetic reserve (Benabid & Bellakhdar, 1987; Bellakhdar, 1997; Chaachouay et al., 2019a). This biodiversity is characterized by considerable floristic endemism, which allows the Rif to occupy a privileged place among the Mediterranean regions with a long medical tradition and traditional know-how based on medicinal and aromatic plants. Indeed, herbal medicine has long played an important role in Moroccan medical practices, and the Rif area is a prime example (Chaachouay et al., 2019b; Chaachouay et al., 2020a).

This investigation aimed to examine medicinal plants growing in the study region to add to indigenous knowledge about MAPs and to analyze the data about existing associations between medicinal species and cardiovascular disorders. Indeed, it is critical to turn this traditional knowledge into scientific knowledge to revalue, maintain, and utilize it properly.

## 2 Methods

### 2.1 Description of the study area

**FIGURE 1:**
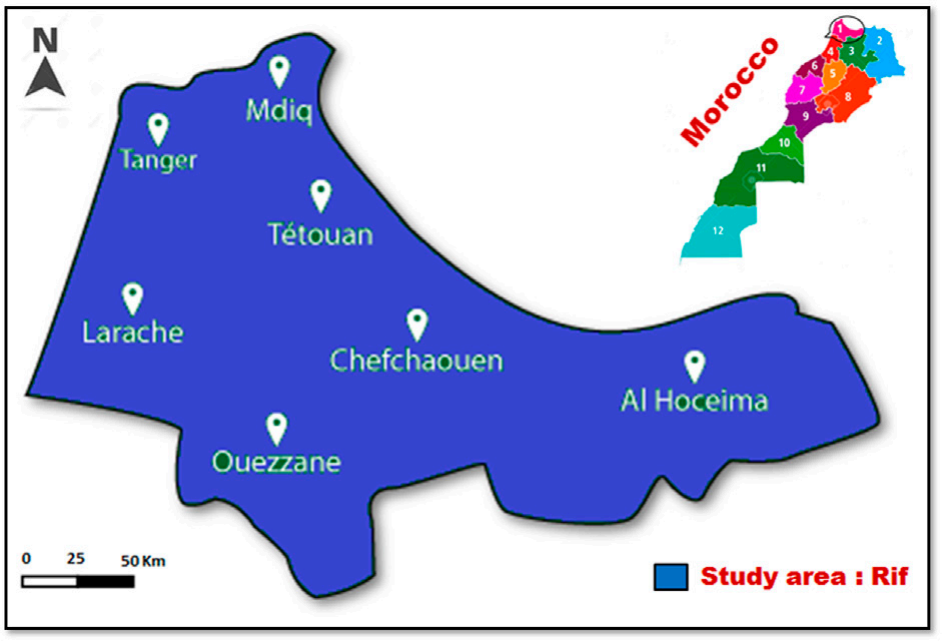
Map of the study area.

The study was conducted in the Rif (northern Morocco). Its northern latitude ranges from 34° to 36°, while its eastern longitude ranges from 4° to 6°. It is bounded to the north by the Strait of Gibraltar and the Mediterranean Sea; to the south by the Rabat-Sale-Kenitra and Fez-Meknes regions; to the east by the Eastern Region the west by the Atlantic Ocean (Figure 1). The general geographical area of the Rif is 11,570 km^2^, and the city’s population is around 3 549 512 inhabitants, with a population density rate of 222.2/km^2^ (HCP, 2018).

The study region has a Mediterranean climate, with the most significant temperature surpassing 45°C during summer (July-August) and below 0°C during winter (December-January). At the same time, the average annual precipitation varies from 700 to 1,300 mm, falling mainly between October and February (DMNM, 2018). It is mountainous, with heights varying from 145 to 2.456 m above sea level (Jbel Tidirhine). This area is dominated by species such as *Cedrus atlantica* (Endl.), *Quercus suber* L, *Quercus ilex* L, *Quercus canariensis* Willd, *Abies marocana* Trab, *Pinus halepensis* Mill*.*, *Juniperus oxycedrus* L, *Ceratonia siliqua* L, and *Pistacia atlantica* Desf. Rif people rely heavily on subsistence farming, cattle, and, to a lesser extent, forest reserves for a living (Benabid & Bellakhdar, 1987; Chaachouay et al., 2019c).

### 2.2 Methodology

#### 2.2.1 Data collection

**FIGURE 2:**
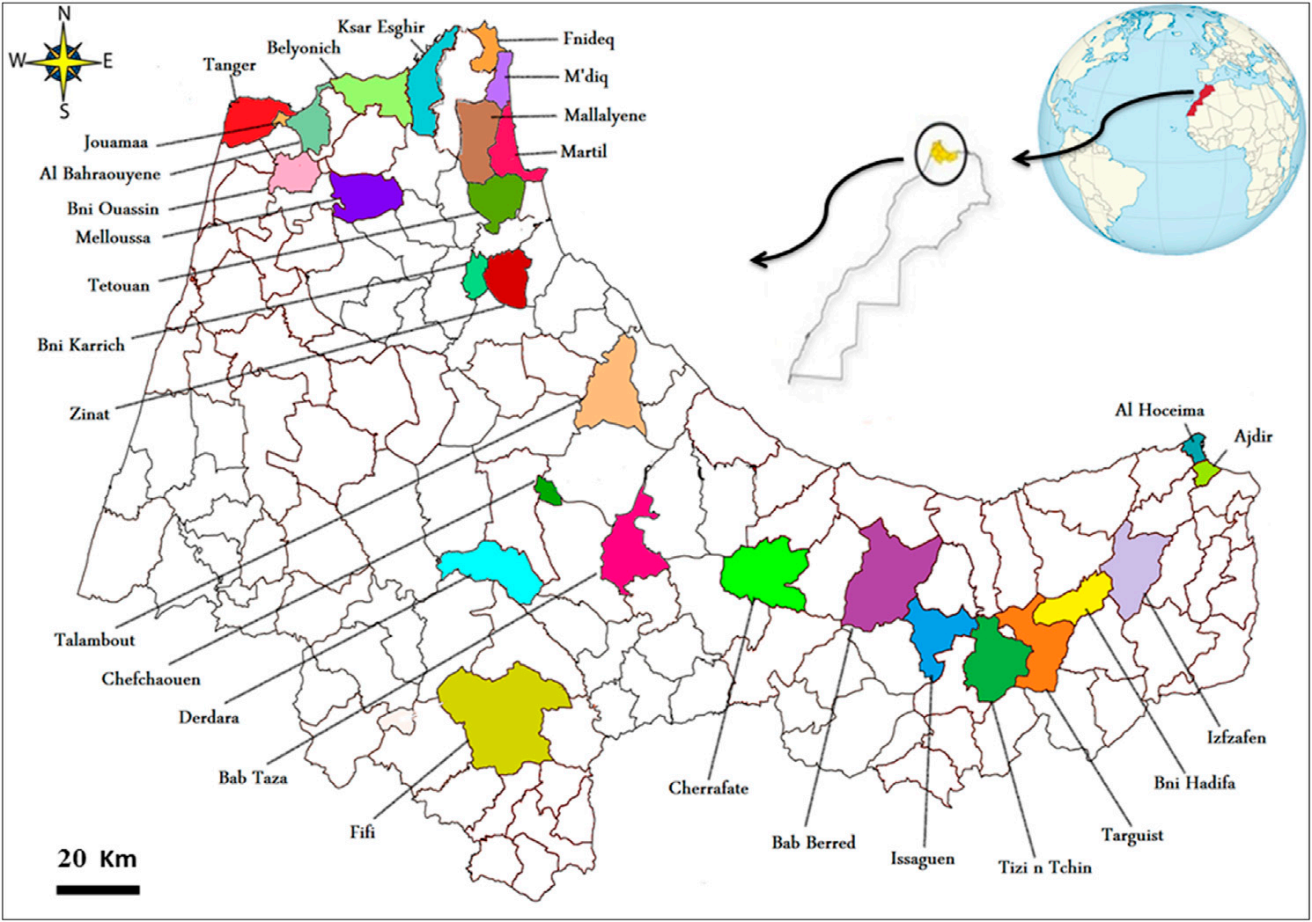
Distribution of survey points at the study area level.

From 30 June 2016, to 1 June 2018, an ethnomedical study was conducted to gather information on plant species used to treat cardiovascular illnesses in the Rif. Semi-structured interviews, open-ended questions, group discussions, free listing, and notes and recordings using a digital voice recorder were used to gather data (Cotton & Wilkie, 1996; Klotoé et al., 2013). Five hundred twenty respondents between the ages of 17 and 81 were selected randomly for conversations (cautery installers, farmers, elders, bonesetters, herbalists, and therapists) in the study region. Following a stratified random sampling procedure, samples were created in each of the 28 strata, including seven urban communes, and then combined to form the total sample of 520 informants (Figure 2).

The number of respondents varies according to the availability of medicinal plants desired in each stratum (Figure 2). Each interview lasted around 20 min to an hour. The interviewee’s profile (age, sex, educational status, monthly salary, family status, and location) and ethnomedicinal statistics for each plant gathered include the common local name, the route of administration, the preparation methods, the dosage, the part used, the condition of the plant used, and the diseases treated Appendix A. The inhabitants of the Rif speak Amazigh or Arabic dialects, and interviews are performed in these languages. All documentation has since been translated into English.

#### 2.2.2 Medicinal plants identification and preservation

We documented the collection of plant materials, then dehydrated, positioned, processed, and conserved plant components (Jain, 1964). Specimens were identified, gathered, dried, compressed, poisoned, and placed on standard herbarium sheets for identification based on ethnomedicinal knowledge supplied by our interviewees. The plants were classified systematically according to their common name, scientific name, family, the plant parts utilized, production method, relative abundance, and geographical distribution. This plant species indicated by the informants were taxonomically identified using local flora available, such as “The medicinal plants of Morocco” (Sijelmassi, 1993), and “Practical flora of Morocco” (Fennane et al., 1998, 1999, 2014), and “Catalog of vascular plants of north Morocco tomes I and II” (Valdés, 2002). Finally, the voucher specimens were placed at the Herbarium, Department of Biology, Ibn Tofail University, Morocco, for future reference.

#### 2.2.3 Data analysis

A descriptive and quantitative statistical technique was applied (ANOVA One-way and Independent Samples *t*-Test, *p*-values of 0.05 or less were considered significant). The findings of the ethnobotanical survey were evaluated using the Family Importance Value (FIV), Relative Frequency of Citation (RFC), Plant Part Value (PPV), Fidelity Level (FL), and Informant Consensus Factor (ICF) (ICF). The Statistical Package for Social Science (SPSS) version 21 and Microsoft Excel 2010 was used for all statistical analyses.

##### 2.2.3.1 *Family importance value*

The FIV recognizes the importance of plant families. It is a cultural significance measure that may be used in ethnobotany to assess the worth of natural plant taxa. To calculate FIV, we use the following formula: $\text{ FIV}=\frac{FC_{family}}{{\text{N}}_{\text{S}}}.$ Where FC_family_ = RFC is the number of informants who mentioned the family, and Ns denotes each family’s total number of species (Sreekeesoon & Mahomoodally, 2014).

##### 2.2.3.2 *Frequency and relative frequency of citation*

The relative frequency of citation (RFC) is calculated by dividing the frequency of citation (FC) by the total number of survey participants (N). The RFC value for medicinal plant species is calculated using the proportion of informants who mention the species. The following formula was used to compute the RFC (Tardío & Pardo-de-Santayana, 2008): $\text{RFC}=\frac{FC}{\text{N}}$ with (0 < RFC <1).

##### 2.2.3.3 *Plant part value*

Plant part value (PPV) was calculated using the following formula: $\;\text{ PPV }=\frac{{\text{RU}}_{\text{Plant part}}}{\text{RU}}\;.$ Where RU is the number of uses reported of all parts of the plant and RU_plant part_ is the sum of uses reported per part of the plant. The section with the greatest PPV is the most often utilized by responders (Chaachouay et al., 2019d).

##### 2.2.3.4 *Fidelity level*

The fidelity level (FL) refers to the proportion of informants who acknowledged using certain plant species to cure a specific condition in the research location. This formula is used to determine the FL index (Friedman et al., 1986): $\text{ FL }\left(\text{\%}\right)=\frac{\text{Np}}{\text{N}}\text{x }100$ . Where Np denotes the number of informants who assert using a particular plant species to cure a specific ailment and N represents the number of informants who claim the use of plants as a medication to treat any given disease.

##### 2.2.3.5 *Informant consensus factor*

An informant consensus factor was derived from seeking an agreement between the informants on the documented cures for each group of diseases (Heinrich et al., 1998). $\text{ICF}=\frac{\text{Nur}-\text{Nt}}{\text{Nur}-1}$ . Nur denotes the total number of usage reports for each illness category, and Nt indicates the number of species utilized.

## 3 Results

### 3.1 Socio-demographic features of the informants

Six hundred local informants were questioned, including 311 females and 289 men (a female/male sex ratio of 1.08). Traditional herbal treatments have an effect on both sexes in the Moroccan Rif. At the research area level, the majority of respondents (45.7 percent) were between the ages of 40 and 60, followed by informants older than 60 years (25.8 percent) and informants between the ages of 20 and 40 years (22.3 percent). Finally, informants less than 20 years old are placed last (6.2 percent). The analysis of the collected data reveals that married people (80.3 percent) use MAPs significantly more than divorced people (10.3 percent). In comparison, widowers account for 7.3 percent of the population. Singles account for only 2.1 percent because married people can avoid or minimize the material charges imposed by the doctor and pharmacist. In terms of education, 75.2 percent of respondents are illiterate, with elementary education accounting for 20.3 percent. Nonetheless, just 4.5 percent of persons with a secondary education utilize medicinal herbs. However, 63.4 percent were jobless, 26.1 percent had a low socioeconomic level, 10% had an average level, and just 0.5 percent had a better income (Table 1).

**TABLE 1:** Sociodemographic details of the informants in the Moroccan Rif area.

Variables	Categories	Number of informants *n* = 600	Percentages (%)	*p*-values
Gender	Female	311	51.8	0.053
	Male	289	48.2	
Age groups	<18 years	37	6.2	0.000
	20–40	134	22.3	
	40–60	274	45.7	
	>60 years	155	25.8	
Family situation	Married	482	80.3	0.000
	Divorced	62	10.3	
	Widower	44	7.3	
	Single	12	2.1	
Educational level	Illiterate	451	75.2	0.000
	Primary	122	20.3	
	Secondary	27	4.5	
	University	0	0.0	
Income/month	Unemployed	380	63.4	0.000
	250–1500 DH	157	26.1	
	1,500–5000 DH	60	10	
	>5000 DH	3	0.5	

### 3.2 Medicinal plant species of the study area

Thirty-three medicinal plant species from 20 botanical families were employed to treat cardiovascular disorders in the study region. Among the information acquired during this study was the local name of the medicinal plants, their scientific name, preparation methods, the portion of the plant utilized, and the medical prescription for which it is employed. The data are organized alphabetically by family name, and the FIV, RFC, FL, and ICF data are shown in Tables 2. According to the number of species and the FIV score, the most botanical family of medicinal plant species used to treat cardiovascular disorders was Poaceae with seven species (FIV 0.02), followed by Fabaceae with three species (FIV 0.035). In comparison, other families had only one or two species.

**TABLE 2:** List of medicinal and aromatic plant actives on the cardiovascular diseases in the Moroccan Rif region.

Family and scientific name	Vernacular name	Part used	Mode of preparation	Medicinal uses	FL	FC	RFC	FIV
Amaranthaceae								0.093
*Spinacia oleracea* L	Sabanikh, Selq	Leaf	Raw	HA	100	56	0.093	
Amaryllidaceae								0.159
*Allium sativum* L	Touma, Tishert	Bulb	Cooked	HA	100	118	0.197	
*Allium porrum* L	Borro	Bulb	Infusion	HA	100	72	0.12	
Apiaceae								0.17
*Daucus carota* L	Khizou	Leaf	Decoction	HA	100	102	0.17	
Araliaceae								0.002
*Hedera hebernica* (G.Kirchn.) Carrière	Louwaya	Leaf	Infusion	BP	100	01	0.002	
Arecaceae								0.076
*Phoenix dactylifera* L	Tmar, Tazdayet	Fruit	Other	HA, AG	78	46	0.076	
Asteraceae								0.017
*Carduus getulus* Pomel	Lssan Maghribi	Leaf	Other	AG	100	01	0.002	
*Cynara scolymus* L	Lqoq	Whole plant	Decoction	HA	100	09	0.015	
Cactaceae								0.031
*Opuntia ficus indica* (L.) Mill	Sbar, Zaâboul	Fruit	Infusion	BP	100	01	0.002	
*Opuntia ficus-barbarica* A.Berger	Hendya	Seed	Decoction	HA	100	36	0.06	
Cannabaceae								0.018
*Cannabis sativa* L	Lkif	Seed	Cataplasm	AG	100	11	0.018	
Dryopteridaceae								0.002
*Dryopteris filix-mas* (L.) Schott	Sarkhs Dakar	Leaf	Decoction	BP	100	01	0.002	
Fabaceae								0.035
*Lens culinaris* Medik	Aaddes	Seed	Cooked	HA	100	48	0.08	
*Medicago polymorpha* L	Fessa	Whole plant	Decoction	AG	100	14	0.023	
*Vicia sativa* L	Guersana	Whole plant	Infusion	BP	100	01	0.002	
Geraniaceae								0.005
*Erodium cicutarium* (L.) L'Hér	Rakma Chokrania	Leaf	Cooked	AG	100	03	0.005	
Iridaceae								0.002
*Gladiolus italicus* Mill	Dalbout Itali	Leaf	Other	AG	100	01	0.002	
Lauraceae								0.105
*Laurus nobilis* L	Wrak Sidnamossa, Rend	Leaf	Decoction	HA, AG, BP	0.89	91	0.152	
*Persea gratissima* C.F.Gaertn	Avocat	Fruit	Cataplasm	HA	100	35	0.058	
Malvaceae								0.002
*Hibiscus sabdariffa* L	Karkadé	Leaf	Decoction	BP	100	01	0.002	
Poaceae								0.02
*Zea mays* L	Dra	Fruit	Decoction	HA	100	08	0.013	
*Phragmites communis* Trin	Kseb	Root	Infusion	HA	100	74	0.123	
*Glyceria fluitans* (L.) R.Br	Aaima	Whole plant	Other	BP	100	01	0.002	
*Miscanthus sinensis* Andersson	Kseb Chinwa	Leaf	Decoction	BP	100	01	0.002	
*Pennisetum setaceum* (Forssk.) Chiov	Dyl Ethaalab	Seed	Decoction	BP	100	01	0.002	
*Hordeum murinum* L	Chaair El FIran	Leaf	Infusion	AG	100	01	0.002	
*Avena barbata* Pott ex Link	Chofan Barri	Whole plant	Raw	AG	100	01	0.002	
Polygonaceae								0.043
*Rumex sanguineus* L	Hommida	Leaf	Decoction	AG	100	26	0.043	
Ranunculaceae								0.003
*Ranunculus bullatus* L	Wden Elhallouf	Root	Decoction	AG	100	02	0.003	
Rosaceae								0.082
*Rubus ulmifolius* Schott	Oualik, Tabgha	Leaf	Raw	HA	100	49	0.082	
Rubiaceae								0.104
*Rubia peregrina* L	Fûwa, Tarubya	Root	Infusion	HA	100	123	0.205	
*Galium aparine* L	Lsak	Leaf	Infusion	AG	100	01	0.002	
Solanaceae								0.001
*Solanum sodomaeum* Dunal	Tfah Lfar	Fruit	Cataplasm	BP	100	07	0.001	

HA: heart arrhythmia; AG: angina; BP: blood pressure.

### 3.3 RFC and FL plant species

To assess the relative relevance of reported medicinal plants, the relative frequency of citation (RFC) was determined based on informants’ citations for certain understudied species, with values ranging from 0.002 to 0.205. Results of this study depicted that *Rubia peregrina* L. exhibited the higher RFC (0.205), followed by the *Allium sativum* L (RFC = 0.197), *Daucus carota* L (RFC = 0.17), and *Laurus nobilis* L (RFC = 0.152). Thirteen plant species have the lowest RFC (RFC = 0.002 each). We computed the FL to find the most often utilized species for each disease group. According to our findings, twenty-two species with the greatest FL of 100% were employed by respondents to treat cardiovascular illnesses (Table 2).

### 3.4 Plant parts used to treat cardiovascular problems

**FIGURE 3:**
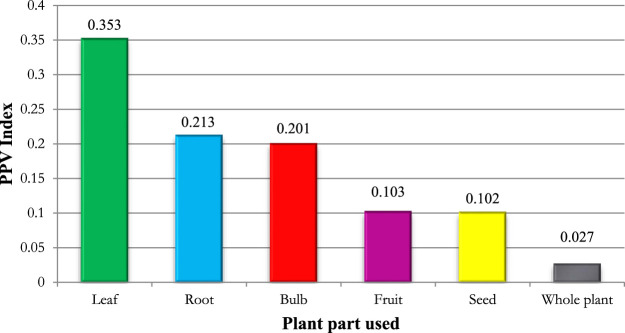
Parts of medicinal plants used in the study area.

The inhabitants of Morocco’s Rif region pick various plant parts to manufacture traditional cures (e.g., seed, root, flower, bulb, fruit, leaf, and whole plant). According to the plant part value PPV index, the leaf was reported as the dominating plant part for cardiovascular treatment manufacture in the research region (PPV 0.353), followed by root (PPV 0.213), bulb (PPV 0.201), fruit (PPV 0.103), seed (PPV 0.102), and the entire plant (PPV 0.027) (Figure 3).

### 3.5 Remedies preparation method and routes of administration

**FIGURE 4:**
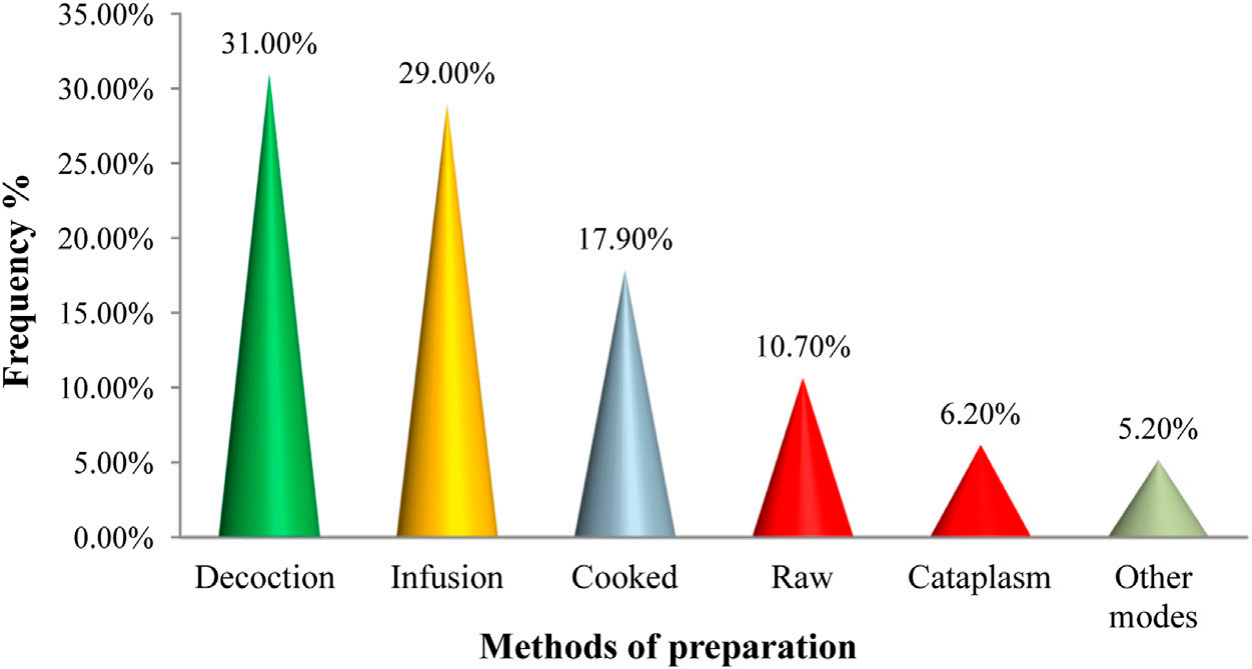
Frequency of different methods of preparation.

Many preparation methods are used to administer the plant’s active ingredients, including decoction, fumigation, cataplasm, infusion, maceration, cooked, and raw (Figure 4). In the study region, decoction continues to be the most common method of preparation (31%), followed by infusion (29%), cooked (17.9%), and raw (10.7%) cataplasm (6.2 percent). The combined proportion of other preparation forms (fumigation, maceration) is not more than 5.2 percent. The method of administration varies depending on the ailment and the ingredients employed. In general, the majority of prepared recipes (84 percent) are administered orally, followed by massage (5.7 percent), various modes of administration (5 percent), swabbing (4 percent), and washing (4 percent) (1.3 percent).

### 3.6 Conditions of medicine preparation

Most of the time, the locals said that they prefer the fresh plant part over the dried plant part for medicine making. The bulk of the cures in the research region (62%) were made from new portions of medicinal plants, followed by dried forms (35.4%) and (2.6%) made from both dry and fresh plant components.

### 3.7 Source of knowledge about medicinal plants

**FIGURE 5:**
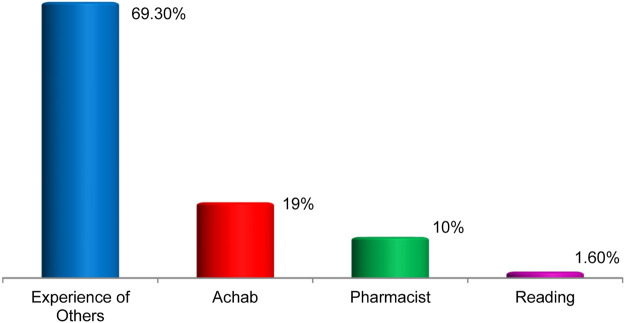
Traditional knowledge acquisition modes.

In our ethnobotanical study, we discovered that 69.3 percent of the population gained information about the therapeutic use of plants as a cure for cardiovascular problems from the experiences of others. This shows the relative transmission of cultural behaviors across generations. 19% of respondents practice herbal medicine on the advice of herbalists, 10.3 percent received information from a pharmacist, and only 1.6 percent gained knowledge through reading books about traditional Arab medicine, watching television programs, or through personal experience with a large number of medicinal plants in their surroundings. As a result, the environment and the experiences of others continue to be the most effective methods of transmitting information about the medical uses of plants (Figure 5).

### 3.8 Medicinal use and informant consensus factor

Cardiovascular illnesses are classified into three ailment groups in the study region, and informant consensus factor (ICF) analyses were performed. The ICF values for the various uses categories in this research varied from 0.44 to 0.98. A total of 33 species have been identified as potential therapeutic agents for cardiovascular disorders. For each class, the informant consensus factors have been determined (Table 3). The most incredible ICF value (0.98) is found for disorders connected with cardiac arrhythmia, whereas the lowest ICF value (0.44) is founded for hypertension.

**TABLE 3:** ICF values by categories for treating cardiovascular diseases.

Categories	List of plant species used and number of citations	Total number of		ICF
		Species	Use citations	
Heart arrhythmia (HA)	*Daucus carota* L. (102), *Allium porrum* L. (72), *Cynara scolymus* L. (9), *Opuntia ficus-barbarica* A.Berger. (*36*)*, Spinacia oleracea* L. (56), *Lens culinaris* Medik. (48), *Laurus nobilis* L. (81), *Persea gratissima* C.F.Gaertn. (35), *Allium sativum* L. (118), Zea mays L. (8), *Phragmites communis* Trin. (74), *Rubus ulmifolius* Schott. (49), *Rubia peregrina* L. (123), *Phoenix dactylifera* L. (10)	14	821	0.98
Angina (AG)	*Phoenix dactylifera* L. (36), *Carduus getulus* Pomel. (1), *Cannabis sativa* L. (11), *Medicago polymorpha* L. (14), *Erodium cicutarium* (L.) L'Hér. (3), *Gladiolus italicus* Mill. *(1), Hordeum murinum* L. (1), *Avena barbata* Pott ex Link. (1), *Rumex sanguineus* L. (26), *Ranunculus bullatus* L. (2), *Galium aparine* L. (1), *Laurus nobilis* L. (7)	12	104	0.88
Blood pressure (BP)	*Hedera hebernica* (G.Kirchn.) Carrière. (1), *Opuntia ficus indica* (L.) Mill. (1), *Dryopteris filix-mas* (L.) Schott (1), *Vicia sativa* L. (1), *Hibiscus sabdariffa* L. (1), *Glyceria fluitans* (L.) R.Br. (1), *Miscanthus sinensis* Andersson. (1), *Pennisetum setaceum* (Forssk.) Chiov. (1), *Solanum sodomaeum* Dunal. (7), *Laurus nobilis* L. (3)	10	18	0.44

## 4 Discussion

Traditional herbal treatments have an effect on both sexes in the Moroccan Rif. However, females have a greater knowledge of the plant species and their use with a predominance of 51.8% against a percentage of 48.2% among males though the test (independent sample *t*-test) did not show a significant difference (*p* = 0.053) between male and female informants on the number of medicinal plant species they listed and associated uses reported. The vigilance of women for the balance the illness and their loyalty to everything traditional might explain this female preponderance. Women provide food and treatment to their families in times of disease. These findings corroborate the findings of earlier ethnobotanical studies conducted nationwide (Chaachouay et al., 2019a; Orch et al., 2021; Benkhnigue et al., 2022).‬‬

The majority of respondents were between the ages of 40 and 60, followed by informants older than 60 years. ANOVA One-way analysis revealed significant differences (*p* = 0.000). The responders of the most significant age offer more accurate information because they possess a large amount of ancestral knowledge that is passed down orally. As a result, there is a loss of ability about MAPs, which may be explained by the skepticism of some young people, who are disinterested in this herbal medication due to modernity and foreign cultural influences. Conventional medical knowledge passed down from generation to generation is in jeopardy since transmission between the elderly and the younger generation is not always guaranteed (Anyinam, 1995). These findings corroborate those observed in other parts of Morocco (Orch et al., 2021; Benkhnigue et al., 2022).‬‬

The analysis of the collected data reveals that married people use medicinal plants significantly more than others. The relationship between family status and indigenous knowledge of cardiovascular disease management was statistically significant (*p* = 0.000). These results corroborate earlier studies done by in others areas (El Hilah et al., 2015). In terms of education, 75.2 percent of respondents are illiterate, with elementary education accounting for 20.3 percent. As a result, the difference in educational level and indigenous knowledge was statistically significant (*p* = 0.000). As a result, the utilization of MAPs diminishes as the degree of study rises. This conclusion is consistent with the results of previous investigations (Lahsissene et al., 2009; El Hilah et al., 2015; Bouzid et al., 2017; Chaachouay et al., 2021). In our study, 63.4% were unemployed, 26.1% had a low socioeconomic level, and 10% had an average level, just 0.5 percent had a better grade. The difference in monthly income and indigenous knowledge was statistically significant (*p* = 0.000). The expensive expense of current medical treatments and their adverse effects are among the primary reasons why respondents preferred herbal therapy. As a result, we can observe that the usage of plants grows as the monthly income of these informants rises. These findings are comparable to those surveyed in Morocco’s Moyen Moulouya (Douiri et al., 2007).

Thirty-three medicinal plant species from 20 botanical families were employed to treat cardiovascular disorders in the Rif. According to the number of species the most botanical family of medicinal plant species used by local people of Rif to treat cardiovascular disorders was Poaceae with seven species (FIV 0.02), followed by Fabaceae with three species (FIV 0.035). In comparison, other families had only one or two species. This large percentage of Poaceae may be explained by the family’s significant presence in the Rif’s flora due to ecological variables that support the growth and adaption of the majority of this family’s species. This representation has been found in various ethnomedicinal studies undertaken in different areas, with minor divergences (Jouad et al., 2001; Eddouks et al., 2002; Okello et al., 2010; Yetein et al., 2013; Ahmad et al., 2014; Orch et al., 2021; Benkhnigue et al., 2022).‬‬

Results of this study depicted that *Rubia peregrina* L. exhibited the higher RFC (0.205), followed by the *Allium sativum* L (RFC = 0.197), *Daucus carota* L (RFC = 0.17), and *Laurus nobilis* L (RFC = 0.152). Thirteen plant species have the lowest RFC (RFC = 0.002 each). These plants had the highest RFC index because many informants cited them, and RFC is directly proportional to the number of informants who mention the usage of a particular plant. Medicinal plant species with a high RFC should be further analyzed phytochemically and pharmaceutically to determine their active ingredients for any medication extraction (Vitalini et al., 2013). These species should also be conserved since their chosen usage may jeopardize their populations owing to overharvesting. According to our findings, twenty-two species with the greatest FL of 100% were employed by respondents to treat cardiovascular illnesses. The most excellent FL score implies that plant species are favored by the research population when treating a specific condition. The MAPs with a high degree of authenticity have more therapeutic potential and include more natural components in the Moroccan Rif area (tannins, flavonoids, and alkaloids).

Concerning the plant part value PPV index, the leaf was reported as the dominating plant part for cardiovascular treatment manufacture in the research region (PPV 0.353), followed by root (PPV 0.213) and bulb (PPV 0.201). The choice of leaves was based on their ease of availability, ease of harvesting, and ease of remedy formulation. Furthermore, the leaves are the site of photosynthesis and, in some instances, the storage of secondary metabolites that contribute to the plant’s biological features. Our findings on the amounts of various plant parts utilized in this study accord with the majority of earlier ethnomedicinal studies conducted in numerous different countries, which have revealed the dominance of the leaf as a material used in the manufacture of herbal medications (Teklehaymanot & Giday, 2007; Lulekal et al., 2008; Simbo, 2010; Chaachouay & Zidane, 2019; Chaachouay et al., 2020b; Chaachouay et al., 2021b; Benkhnigue et al., 2022).

In the study region, decoction continues to be the most common method of preparation, followed by infusion, and cooked. The combined proportion of other preparation forms (fumigation, maceration) is not more than 5.2 percent. The decoction’s widespread usage is explained by the fact that it allows for the collection of the most active elements and attenuates or eliminates the poisonous impact of some recipes. In other parts of Morocco, ethnobotanical research surveys revealed that most respondents prepared the treatment using decoction (Ramana, 2008; Ghasemi et al., 2013; Chaachouay et al., 2020a; Chaachouay & Zidane, 2021; Kumar et al., 2021; Orch et al., 2021; Benkhnigue et al., 2022). This demonstrates that the Moroccan people are constantly exchanging knowledge about the usage of therapeutic and aromatic herbs. At the continental level, decoction is described as the primary preparation technique (Okello et al., 2010; Stangeland et al., 2011; Yetein et al., 2013). The majority of prepared recipes are administered orally, followed by massage. The region’s high frequency of internal diseases may explain the region’s preponderance of oral administration (Polat & Satıl, 2012). On the other hand, it is assumed that the oral route is the most agreeable to the patient. The preponderance of oral administration of several medicinal plants in Moroccan Rif is entirely consistent with most ethnobotanical investigations conducted in Africa (Abdurhman, 2010, 2010; Benarba et al., 2014; Sreekeesoon & Mahomoodally, 2014; Chermat & Gharzouli, 2015; Chaachouay et al., 2021a; Chaachouay & Zidane, 2022).‬

The bulk of the cures in the research region (62%) were made from fresh parts of medicinal plants. According to research done in Tigray (Abdurhman, 2010) 86 percent of preparations were in a fresh form, whereas Getahun (Getahun, 1976) revealed that 64 percent of medicinal herbs were utilized in fresh condition and 36 percent in dried form. The Moroccan Rif people’s reliance on fresh materials is mainly owing to the efficacy of fresh medicinal herbs in therapy since the contents are not lost before usage, as opposed to dried versions.

The ICF values for the various uses categories in this research varied from 0.44 to 0.98. A total of 33 species have been identified as potential therapeutic agents for cardiovascular disorders. For each class, the informant consensus factors have been determined. The most incredible ICF value (0.98) is found for disorders connected with cardiac arrhythmia, whereas the lowest ICF value (0.44) is founded for hypertension. The ICF findings showed that illnesses prevalent in the Moroccan Rif region had a higher informant consensus factor (0.98). These high ICF values suggested that informants were reasonably reliable on the usage of medicinal plant species (Lin et al., 2002). The informant consensus values also suggested that the people share knowledge about the most important medicinal plant species for treating the most often encountered ailments in the research region. As a result, species with high ICF should be prioritized for future pharmacological, toxicological, and phytochemical investigations.

## 5 Conclusion

The ethnobotanical survey found that the study region is rich in biodiversity, with a diverse range of medicinal and aromatic species, and that more inquiry is necessary. This opulent flower arrangement demonstrates the enormous potential for traditional knowledge to aid in creating natural product derivatives as inexpensive medications. These plants continue to play a critical part in people’s lives in the Moroccan Rif. However, ethnomedicinal data for medicinal plants used to treat cardiovascular disorders in this area is lacking. According to the findings of this study, increasing the use-value, preference ranking scores, and fidelity level values of recorded medicinal plant species will facilitate future pharmaceutical and phytochemical research and conservation methods. In this regard, emphasis should be placed on conserving traditional medicinal plants and related indigenous knowledge in the Moroccan Rif region to ensure their continued viability.

## Data Availability

The original contributions presented in the study are included in the article/Supplementary Materials, further inquiries can be directed to the corresponding author.
